# Dissecting the genetic basis of drought responses in common bean using natural variation

**DOI:** 10.3389/fpls.2023.1143873

**Published:** 2023-09-14

**Authors:** Diana Labastida, Pär K. Ingvarsson, Martha Rendón-Anaya

**Affiliations:** ^1^ Linnean Centre for Plant Biology, Department of Plant Biology, Uppsala BioCenter, Swedish University of Agricultural Science, Uppsala, Sweden; ^2^ Department of Biological and Environmental Sciences, University of Gothenburg, Göteborg, Sweden

**Keywords:** drought, GWAS, stay-green, common bean (*Phaseolus vulgaris* L), crop resilience

## Abstract

The common bean (*Phaseolus vulgaris* L) is the most important legume for human consumption, contributing 30% of the total daily protein intake in developing countries. A major limitation for its cultivation is drought, which causes more than 60% of the annual losses. Among physiological adaptations to drought, delaying senescence and extending the photosynthetic capacity can improve crop productivity. This strategy is known as functional “*stay-green*” (SG) and has been discussed as a goal in plant breeding to alleviate the loss of yield under water scarcity conditions. The genetic components behind SG traits have been explored specially in cereals, but they are to date poorly studied in the common bean. For this, we screened 71 common bean cultivars belonging to the three most important gene-pools, Mesoamerica, Andes and Europe, selected to cover the natural variation of the species. Phenotyping experiments under terminal drought during long-days in greenhouse conditions, identified six photoperiod insensitive cultivars of European origin with a clear SG phenotype. Using SNP data produced from whole genome re-sequencing data, we obtained 10 variants significantly associated to the SG phenotype on chromosomes 1, 3, 7, 8, 9 and 10 that are in close proximity to gene models with functional annotations related to hormone signaling and anti-oxidant production. Calculating pairwise F_ST_ between subgroups of cultivars divided according to their drought response (susceptibility, escape, recovery or SG), we identified up to 29 genomic windows accounting for 1,45Mb that differentiate SG cultivars; these signals were especially strong on chromosomes 1, 5 and 10. Within these windows, we found genes directly involved in photosynthetic processes and trehalose synthesis. Altogether, these signals represent good targets for further characterization and highlight the multigenic nature of the SG response in legumes.

## Introduction

Fluctuations in precipitation are a natural part of the climate cycle but because of recent climate change, droughts are becoming more frequent, severe, and pervasive. This in turn has impacted crop production, especially in arid and semi-arid areas (Jha et al., 2019) and thus, understanding the physiological and genetic connections between crop yield and water requirements is essential to develop more precise and appropriate adaptation strategies. Legumes play a fundamental role in food security in developing countries. Within this group, the common bean, *Phaseolus vulgaris* L, is especially important for human consumption in terms of nutrients provided: dry beans contain up to 22% of protein, essential nutrients as calcium, iron, magnesium, phosphorus, potassium, as well as complex carbohydrates (62%) and soluble fiber (15%) (Myers and Kmiecik, 2017). Drought stress is one of the most limiting abiotic factors to bean cultivation, affecting up to 60% of worldwide production, and it is the second largest cause of yield loss after diseases (Villordo-Pineda et al., 2015). This is especially true for developing countries where it is often cultivated by small farmers and hence depends on natural rainfall (Mukeshimana et al., 2014). Water stress also decreases seed mineral nutrients and affects N_2_ fixation in legumes (Smith et al., 2022).

Drought episodes have been defined as the inadequacy of water availability in quantity and distribution during the life cycle of the crop, and they depend on precipitation and soil moisture storage capacity (Beebe et al., 2013). Drought stress can also occur even if water is not scarce, for example in saline environments and in soils with temperatures between 0-15°C (Rosales-Serna et al., 2004). Drought can be categorized into different types based on the time point of the crop growth cycle when it occurs: if water shortage occurs during the first two weeks after planting it is defined as early drought; if there are short periods of drought within the entire phenological cycle, it is called intermittent drought; but if it occurs during the reproductive stage, it is called terminal drought (Rosales et al., 2012). This type has the most detrimental consequences for farmers as it affects grain filling and seed yield.

Plants have developed different physiological mechanisms of adaptation to drought that are often grouped into the following categories: drought escape, drought avoidance, drought tolerance and recovery, although they are not mutually exclusive (Rosales-Serna et al., 2004). Drought escape occurs when the plant can complete its life cycle before severe water deficits, which involves early flowering, plasticity in the duration of the growth periods and remobilization of photosynthates to the grains (Beebe et al., 2013). Drought avoidance is the ability of the plant to maintain high tissue water potential, often achieved by efficient root systems. Drought recovery can be defined as the ability of the plant to recover its greenness after a period of drought. Finally, drought tolerance is defined as the capacity of a plant to cope with water deficit with low tissue water potential through osmotic adjustments and increasing cell elasticity (Beebe et al., 2013; Ilyas et al., 2020). At the cellular level, the decrease in the water potential of plant tissues triggers a variety of processes, such as growth inhibition, accumulation of abscisic acid and osmo-protecting solutes, the production of reactive oxygen species (ROS), oxidation of proteins and lipids, stomatal closure, reduced transpiration and photosynthetic rates, formation of radical scavenging compounds, and changes in the accumulation levels of proteins and small RNAs (Rosales et al., 2012).

In the common bean some morphological adaptations to drought that have been observed include the loss of leaf area which results from a series of events such as the reduction in size of the younger leaves, or accelerate leaf loss by senescence [reviewed by (Beebe et al., 2013)]. Senescence in annual crop plants overlaps with the reproductive phase, however, when it occurs prematurely under stress conditions such as drought, it severely reduces crop yield. Conversely, cases of delayed senescence that extend the photosynthetic period, even under non-drought conditions, have been associated to drought tolerance and higher yields under water-limiting conditions (reviewed by (Gregersen et al., 2013)). This delayed senescence and impaired chlorophyll catabolism are known as “stay*-green*” (SG) traits. Consequently, SG plants have been discussed as one potential goal to increase crop productivity, particularly when exposed to abiotic stress. Studies on the genetic basis of the SG phenotype and its relationship to drought tolerance have highlighted candidate genes in species such as corn (Bengoa Luoni et al., 2019), sorghum (Rama Reddy et al., 2014; Johnson et al., 2015), and chickpea (Sivasakthi et al., 2019). In rice, reports have documented differences at the promoter region of the *Stay-Green* gene (encoding a chlorophyll-degrading Mg++-dechelatase, OsSGR) between the *japonica* and *indica* subspecies, that modulate the onset of senescence and with that, alter their photosynthetic competence and grain yields (Shin et al., 2020).

The genomic paths behind drought responses have not been easy to elucidate as such traits are typically controlled by various small- effect QTLs in combination with environmental interactions (Jha et al., 2019). GWAS and QTL mapping strategies in common bean populations have identified a large number of genomic regions and gene candidates behind a series of morphological traits under drought stress; for example, Mendes and collaborators (Valdisser et al., 2020) reported 18 QTLs and 35 genes associated to seed weight under drought conditions in the Mesoamerican gene pool. Another study in the Andean and middle-American gene-pools identified 68 SNPs that were significantly associated with key agronomic and physiological traits under drought stressed and well-watered conditions (Mutari et al., 2023).

Despite these large phenotypic screenings, we have overlooked the potential of SG traits in common bean to cope with drought stress and thus, our understanding of the genetic basis of SG in the species is very limited. To gain insights into this strategy, we studied the phenotypic responses to terminal drought in a panel of 71 P*. vulgaris* accessions that cover the most important gene-pools: Mesoamerica, Andes, and Europe. Our classification of drought responses was based on the final yield loss, greenness kept during the experiment as well as the ability of the plants to produce new trifoliate leaves and re-start the reproductive phase once irrigation started again. We identified European accessions with a clear SG phenotype while subjected to terminal drought. Through GWAS and population genetics summary statistics, we identified chromosomal regions and candidate genes behind drought response strategies which represent excellent candidates for further studies and breeding protocols.

## Materials and methods

### Plant material

We used a set of 71 *Phaseolus vulgaris* accessions, selected to span the range of natural variation in the species, that were provided by the International Center of Tropical Agriculture (CIAT), the Leibniz Institute of Plant Genetics and Crop Plant Research (IPK) and NordGene seed banks. This collection includes accessions from three main genetic pools in common bean, the European, the Mesoamerican and the Andean (**Table 1**).

**Table 1:** *Phaseolus vulgaris L.* cultivars considered in this study.

	Accession ID	Genepool	Country of origin	Strategy		Accession ID	Genepool	Country of origin	Strategy
1	NGB18415	EU	Sweden	SG	39	PHA1139	EU	Hungary	R
2	NGB9300	EU	Norway	SG	40	PHA13035	EU	Italy	R
3	PHA1077	EU	Belgium	SG	41	PHA13099	EU	United Kingdom	R
4	PHA2682	EU	Sweden	SG	42	PHA167	EU	Greece	R
5	PHA366	EU	Italy	SG	43	PHA5989	EU	Romania	R
6	PHA6155	EU	United Kingdom	SG	44	G23578A	MA	Colombia	R
7	NGB13468	EU	Sweden	S	45	G3296	MA	Mexico	R
8	NGB23857	EU	Denmark	S	46	G13955	A	Argentina	NA
9	NGB23934	EU	Sweden	S	47	G16843	A	Peru	NA
10	NGB24038	EU	Sweden	S	48	G21043	A	Argentina	NA
11	PHA1086	EU	Belarus	S	49	G19898	AW	Argentina	NA
12	PHA12934	EU	Italy	S	50	G21201	AW	Argentina	NA
13	PHA13228	EU	Slovakia	S	51	G23426	AW	Peru	NA
14	PHA13609	EU	Switzerland	S	52	G23455	AW	Peru	NA
15	PHA13736	EU	United Kingdom	S	53	PHA1753	EU	Romania	NA
16	PHA13960	EU	Spain	S	54	PHA725	EU	Italy	NA
17	PHA14278	EU	Austria	S	55	PHA7686	EU	Romania	NA
18	PHA1772	EU	Slovakia	S	56	PHA99	EU	Greece	NA
19	PHA3673	EU	Austria	S	57	G12865	MW	Mexico	NA
20	PHA4008	EU	Italy	S	58	G12947	MW	Mexico	NA
21	PHA419	EU	Switzerland	S	59	G24323	MW	Mexico	NA
22	PHA5866	EU	Italy	S	60	G14629	EU	Sweden	E
23	PHA6011	EU	Romania	S	61	G8658	EU	Sweden	E
24	PHA6389	EU	Romania	S	62	NGB23936	EU	Sweden	E
25	PHA7150	EU	Spain	S	63	PHA1076	EU	Hungary	E
26	G13094	MA	Mexico	S	64	PHA1137	EU	Hungary	E
27	G4383	MA	Mexico	S	65	PHA1138	EU	Hungary	E
28	G23556	MW	Mexico	S	66	PHA1142	EU	Hungary	E
29	G7930	A	Argentina	R	67	PHA13666	EU	United Kingdom	E
30	PHA6437	EU	Spain	R	68	PHA13928	EU	Switzerland	E
31	PHA7309	EU	Poland	R	69	PHA4534	EU	Hungary	E
32	PHA7313	EU	Poland	R	70	PHA4620	EU	Hungary	E
33	G1282	EU	Sweden	R	71	PHA49	EU	Sweden	E
34	G5340	EU	Sweden	R	72	PHA5934	EU	Albania	E
35	NGB17826	EU	Sweden	R	73	PHA6066	EU	Italy	E
36	NGB20124	EU	Denmark	R	74	PHA6254	EU	Albania	E
37	NGB23858	EU	Norway	R	75	G11015	MA	Mexico	E
38	PHA1022	EU	Poland	R					

MA, Mesoamerican domesticated; MW, Mesoamerican wild; A, Andean domesticated; EU, European. Accession numbers.

The seed banks where the material was obtained are the International Center for Tropical Agriculture, Colombia (CIAT; accession ID: Gxxx), the Nordic Genetic Resource Center (NordGen; ID NGBxxx) and European Search Catalogue for Plant Genetic Resources at Gatersleben, Germany (IPK; ID: PHAxxx).

NA, not assigned.

### Phenotypic data and growth conditions

We screened the panel of 71, unrelated, common bean cultivars under terminal drought stress. This experiment was done between the months of May and July at the Swedish University of Agricultural Sciences in Uppsala, under green-house (GH) conditions. Because of the natural light regime around that time of the year at Scandinavian latitudes (59°N), we needed to make sure the selected accessions were capable of flowering under long days. For this reason, our screening could not include more cultivars from the Americas, that only flower under short/neutral day lengths. The drought treatment started at the reproductive stage labeled R6, that corresponds to the point when the first flower opens (Fernández de et al., 1986). The conditions for this experiment in the GH were: temperature ranging between 25-28° C, 50% humidity and a photoperiod of ~16hr light/8hr darkness. The plants were sown in medium pots (10 cm of diameter) with 750 gr of soil and organized in two experimental blocks, well-watered and water-stressed conditions. The day before the dry-down was initiated, all pots were abundantly watered to reach the saturation point, that refers to when all spaces in the soil are filled with water and allowed to drain overnight. Later, we measured the humidity with a kit W.E.T. Sensor HH2 Moisture Meter Delta-T^®^ ensuring it was at least 35% at the starting point. After two weeks of drought treatment, the plants were re-watered, and the phenotypic traits were measured weekly.

The phenotypic data that allowed us to classify the accessions per drought strategy were number of pods and seeds per plant (produced weekly and at the final harvest). The percentage of yield loss or gain was measured in terms of the number of seeds produced in comparison with the control. We used the formula as it follows:


$$\text{Yield loss }=\frac{\left(control-treatment\right)}{control}x100$$

Plant responses to drought were classified according to their performance during the treatment. Stay-green accessions were identified based on the maintenance of greenness in both stems and leaves throughout the treatment. Escaping cultivars increased the production of pods in response to stress and had a yield loss inferior to 75%. Recovery was assigned to accessions that recovered greenness, produced new trifoliate leaves and/or restarted the reproductive stage once irrigation was reestablished.

### Population structure and differentiation

Whole genome re-sequencing data was produced for the entire collection of *P. vulgaris* accessions. Using SNP data (Rendón-Anaya et al., 2023) we performed population structure analyses in 85 selected accessions: we considered the 71 accessions screened for drought, plus another wild 14 accessions of Mesoamerican and Andean origin, in order to have an accurate reconstruction of the structure of the populations. SNPs were called with GATK (v3.8, Rendón-Anaya et al., 2023); we extracted biallelic sites only, that were pruned for LD, minor allele frequency and missingness of genotypes per sample (plink –maf 0.05 -indep-pairwise 100 10 0.2 -geno 0.1). In total, 126,111 pruned sites along the 11 chromosomes were used to produce a PCA with PLINK (plink –pca; v1.90b4.9) (Purcell et al., 2007).

Pairwise F_ST_ between batches of accessions grouped according to their drought response was calculated on each chromosome in the *Phaseolus vulgaris* genome in 50kb, non-overlapping genomic windows, using the python popgen pipeline available at https://github.com/simonhmartin/genomics_general. For this screening, we used all SNPs on each chromosome that passed the following criteria: min/max sequencing depth of 8 and 25 respectively and that were present in at least 70% of the accessions (vcftools –min- meanDP 8 –max-meanDP 25 –max-missing 0.7) (Danecek et al., 2011).

### Genome-wide association studies

We used the SNP panel of 126,111 sites to run genome-wide association analyses. We converted the vcf file to a matrix of 0,1 and 2 values for homozygous (ref/alt) or heterozygous genotypes with vcftools (vcftools –012). As we allowed 10% of missingness in the initial genotype filtering, we had to impute the missing genotype information to avoid numeric biases in the GWAS calculations. For each column in the matrix representing individual positions, we calculated the mean genotype value (not including missing genotypes coded as -1) that we used to fill the missing genotypes. This numeric matrix was the input for GWAS analyses.

We coded the phenotypic data as binary matrices, i.e. accessions classified under a particular drought strategy were coded with 1, whereas those without that specific response were coded with a 0. GWAS was performed using two models: multiple loci mixed model (MLMM), and Bayesian-information and linkage-disequilibrium iteratively nested keyway (BLINK), all implemented in GAPIT3 (Wang and Zhang, 2021). We controlled for the effects of population structure by setting the number of relevant PCs at 4; we used SNPs with a minimum allele frequency of 0.05. The *p*value threshold was set at an alpha of 0.05 with Bonferroni correction (0.05/number of markers) to determine the significant associations.

## Results

### Drought stress responses

**Figure 1:**
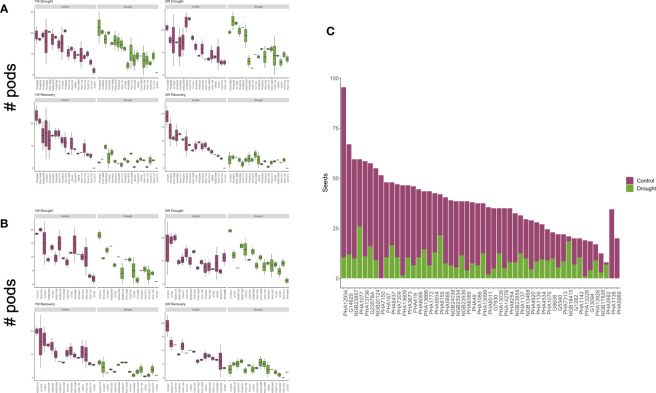
Yield summary. Weekly measure of pod production per accession compared to the control plants during the treatment. Escaped cultivars are shown in **(A)** and recovered in **(B, C)**. Number of seeds produced per accession that were used to classify the drought responses.

Once germinated, the plants took between 25 and 30 days to transition from vegetative to reproductive phases and reach the flowering R6 developmental stage. After two weeks of severe drought stress and yield assessment, we were able to classify the cultivars according to their drought tolerance strategy. As the first response to cope with drought, we observed that several plants accelerated their phenological process, increasing the number of pods produced especially during the first week of stress (**Figures 1A, B**). However, we observed a high percentage of pod abortion (**Supplementary Figure 1**). Those accessions that followed this behavior and managed to complete their cycle with yield losses below 75% were considered drought-escaping. Once the two-week drought treatment finalized, the plants were re-watered until the soil reached ~35% moisture. Some cultivars recuperated their greenness, produced new trifoliate leaves and even re-started pod production.

Grain yield was calculated in terms of seeds per plant at the final harvest (**Figure 1C**). As expected, based on previous observations in common bean, most genotypes suffered substantial yield loss under drought conditions. For example, cultivars PHA4008, PHA1086, PHA419, PHA12934, PHA13609, PHA14278, and PHA3673 were the least productive, with yield losses exceeding 85%. In total, 22 accessions were considered susceptible (**Table 1**), since they suffered yield loss exceeding 75% (**Supplementary Figures 1, 2**) or they were dead after the two-week treatment. Only six accessions under drought stress produced more seeds than the controls in the final harvest; interestingly three of them showed SG traits while the other three were classified as drought-escaping cultivars. Cultivars G1282, PHA13099, PHA167, PHA4534, PHA1137 and PHA13666 had yield losses of less than 35% and although they did not display any SG trait, they might be a good alternative for breeding programs aimed at breeding for drought tolerance.

**Figure 2:**
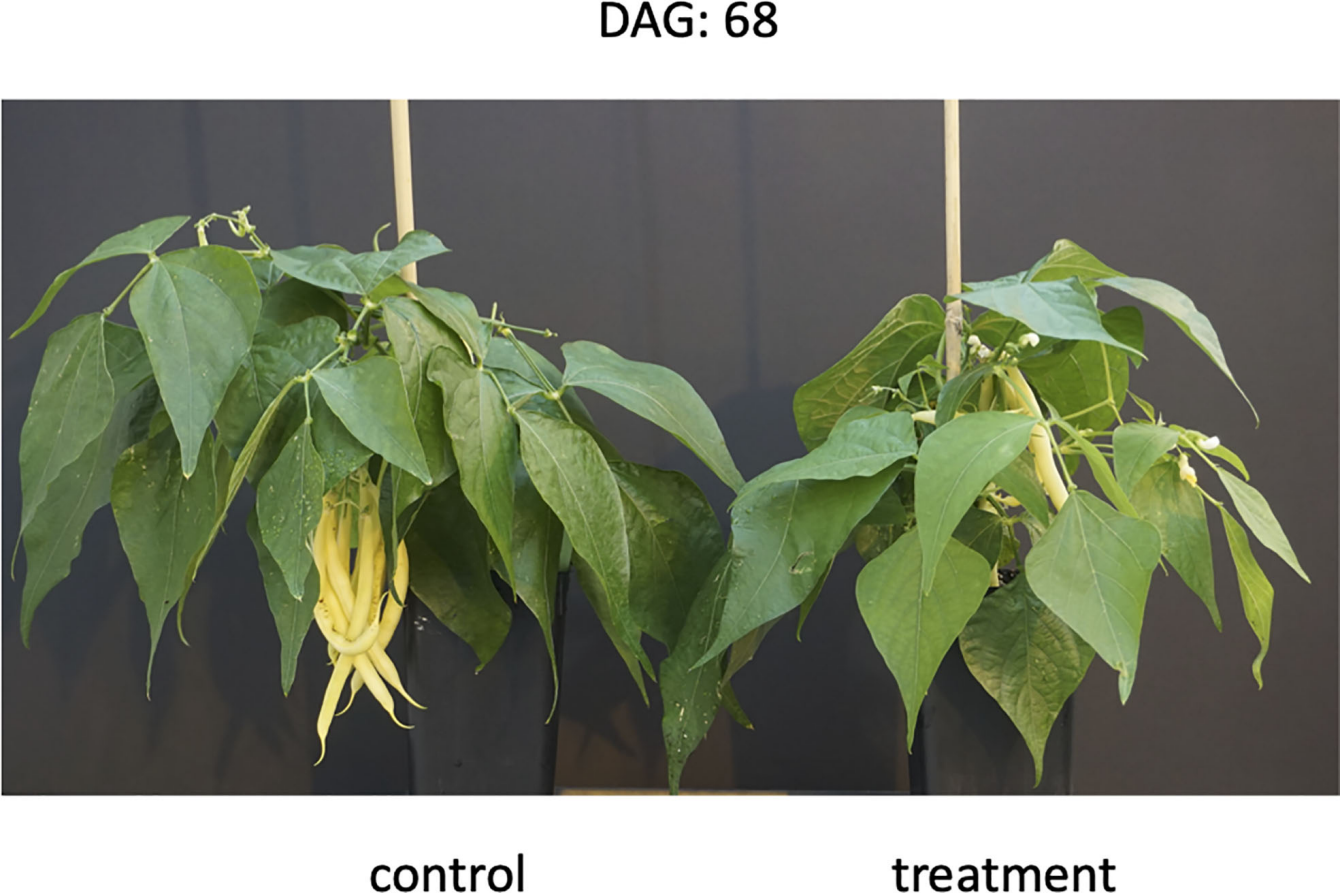
Stay green phenotype observed in the European cultivar PHA6155 at the end of the treatment (drought and recovery), 68 days after germination (DAG).

In total, we observed six SG cultivars that maintained their greenness in both stem and leaves during the whole treatment (**Figure 2**; **Supplementary Figure 2**), 16 escaping drought and 17 that recovered after irrigation re-started (**Table 1**).

### Population structure

**Figure 3:**
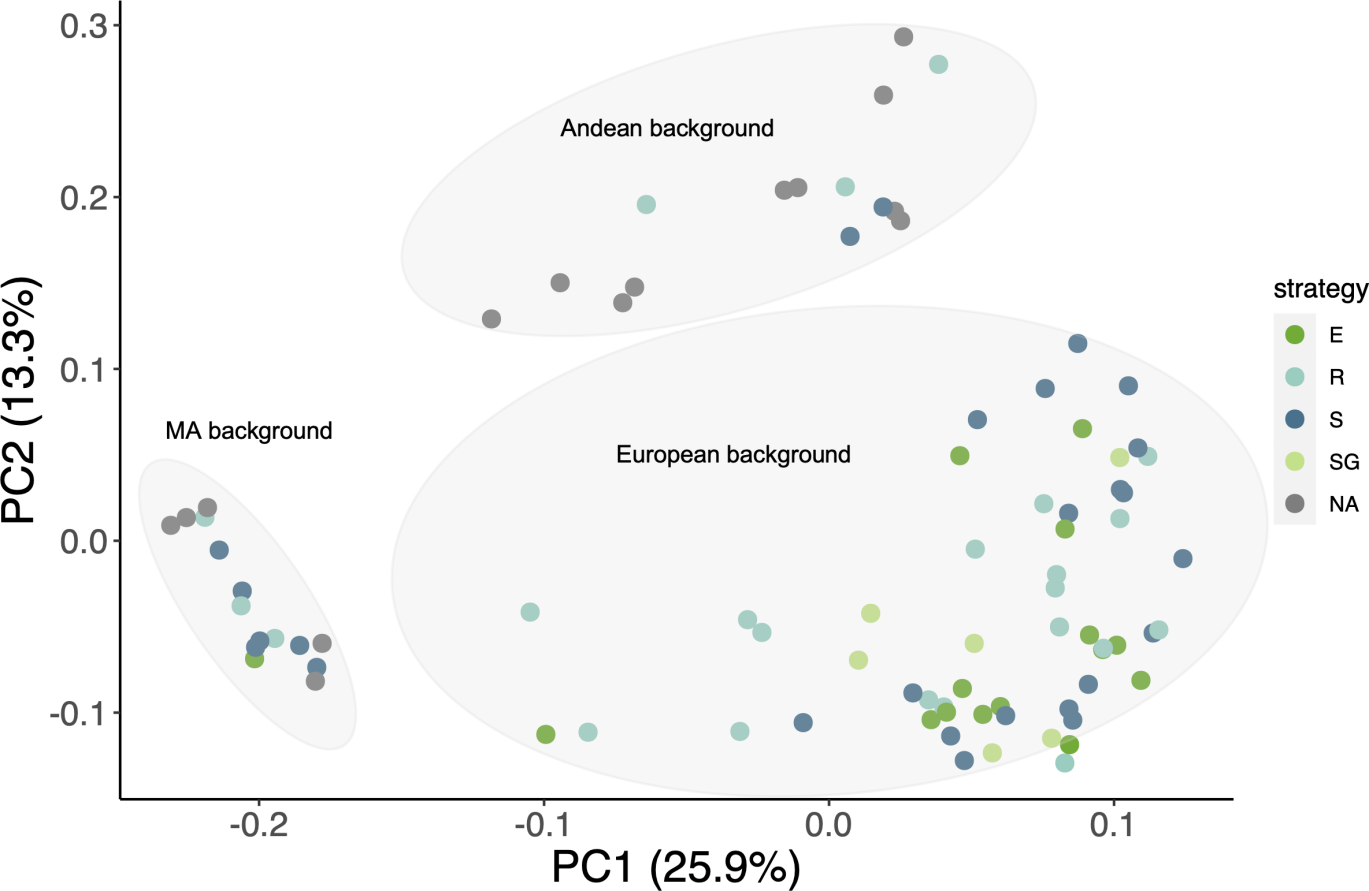
Population structure. SNP-based PCA of the phenotyped cultivars (126,111 pruned sites across the 11 chromosomes). Drought response: E=escape, SG=stay-green, R=recovery and S= susceptible.

A SNP-based principal component analysis was carried out to understand the population structure in our panel of common bean accessions, as well as to assess the distribution of the drought responses across the gene pools (**Figure 3**). Consistent with the sites of collection, the accessions were grouped according to their Mesoamerican (MA), Andean (A) or European (EU) origin, the latter represented by a large cluster that comprises apparent hybrid individuals between MA and A, whereas others display a clear, almost intact MA or A genetic background (**Supplementary Figure 3**). Although most of the accessions that showed some type of drought resistance have a European origin (due to a bias in the number of EU samples considered from the start point), drought tolerance is present in all three gene pools, Interestingly, all accessions identified as SG belong to the EU gene pool.

### Differentiation

**Figure 4:**
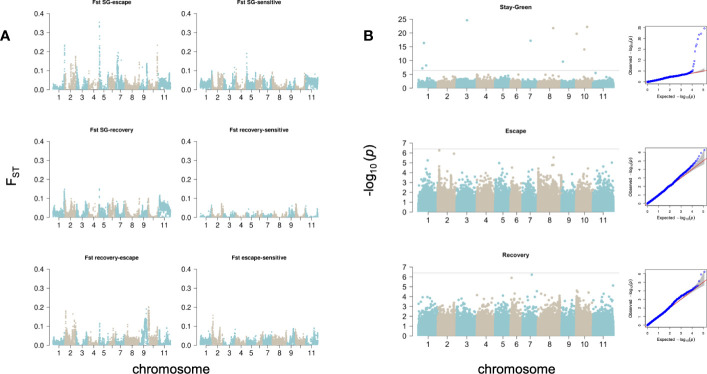
Genomic signals behind drought responses. **(A)** Pairwise F_ST_ was calculated in 50kb non-overlapping windows across the genome. **(B)** GWAS. Manhattan and QQ plots show the per-site *p*-value obtained with Blink for each drought response strategy. The horizontal lines represent the significance threshold (-log_10_(*p*-value)=6.4).

We calculated pairwise F_ST_ in 50Kb windows between the subpopulations obtained after classifying the bean cultivars according to their response to drought, stay-green (SG), escape (E), recovery (R) and susceptible (S). The genome-wide average between strategies were: $\bar{x}$ _(SG vs E)_=0.009, $\bar{x}$ _(SG vs R)_= 0.013 and, $\bar{x}$ _(E vs R)_= 0.014, and between strategies and susceptible accessions. $\bar{x}$ _(SG vs S)_=0.0125, $\bar{x}$ _(E vs S)_= 0.011 and, $\bar{x}$ _(R vs S)_< -0.001 (**Figure 4A**).

We compared the F_ST_ outliers (top 1%) between drought-response subgroups to identify those chromosomic windows that would specifically differentiate each strategy from the rest at the genomic scale. The intersection of F_ST_ outliers between pairwise comparisons SG-E, SG-R and SG-S revealed two regions that differentiate SG varieties in Chr01 (50.3-50,85Mb) and Chr05 (550-750Kb), accounting for 550,000 bp of the genome. The intersection of SG-E and SG-R only added a short region in Chr08 (62.9-63Mb), and the intersection between SG-E and SG-S revealed other regions on Chr02 (26.2-26.35Mb, 27.55-27.6Mb, 46.8-47Mb) and Chr05 (1,35-1,5Mb and 1,75-2Mb). The strongest differentiation was observed between SG and E cultivars, where F_ST_ values reached 0.35 (**Figure 4A**). As most SG and E cultivars are European, we do not expect this strong differentiation to be explained by differences in genetic background (Mesoamerican or Andean) of the cultivars. Similarly, the intersection of F_ST_ outliers identified one single window in Chr02 (35.25-35.3Mb) specific to the E subgroup, while the intersection of E-S with E-R outliers revealed windows on Chr02(3.9-4.4Mb), Chr09 (29.2-30.2Mb) and Chr10(150-400Kb; 1,15-1.6Mb and 2,25-2.3Mb).

### GWAS

We ran genome wide association analyses using filtered, unlinked SNPs and the classification in subgroups of the *P. vulgaris* cultivars as binary phenotypic traits (1/0 denoting presence/absence of each drought strategy). We used multi-locus models, MLMM and Blink implemented in GAPIT3; the advantages of these models in terms of statistical power vs computational cost have been discussed elsewhere (Wang and Zhang, 2021).

Significant sites were identified after correcting for population stratification at a threshold of *p*-value<4e^-7^. On SG accessions we identified outlier SNPs on chromosomes 1,3,7,8,9 and 10 (**Table 2**); in particular, the site Chr07_20981178_A_G was identified as significantly associated to the SG phenotype both with BLINK and MLMM (unique significant site identified with MLMM), with PVE of 8.3% on BLINK and 41% with MLMM. On E accessions we detected a weaker association on chromosome 2 that did not reach the significance threshold (Chr02_3941363, *p*-value 5.5e-07); however, it should be highlighted that this SNP overlaps F_ST_ outlier windows intersecting E-R and E-S, which suggests this region harbors important gene models behind this drought response. Finally, we recovered significant associations with MLMM to R accessions on chromosomes 7 and 8, explaining 41 and 10.3% of phenotypic variation.

**Table 2:** SNPs and gene models identified through GWAS.

Chr	Position	P-value	maf	PVE(%)	Distance to closest gene models	A.thaliana homologue	Annotation
3^a^	27301566	2,38E-25	0,471	30,13	Intergenic 1.9Kb downstream of putative protein (blastx hit, no gene model)	NA	NA
8 ^a^	40863737	1,70E-22	0,050	15,77	Intergenic >50KB from Phvul.008G151830 Phvul.008G151824	AT1G08610 AT3G04760	Pentatricopeptide repeat (PPR) superfamily proteins, chloroplastic
10 ^a^	1252638	1,94E-20	0,064	10,15	Intergenic, 2Kb downstream of Phvul.010G008800 (1254077.1260168)	AT2G18950, HPT1	Involved in the synthesis of tocopherol (vitamin E)
7 ^a^	20981178	6,62E-18	0,429	8,33	Intergenic, 10Kb upstream of Phvul.007G134000	AT2G37060, NF-YB8	Component of the NF-Y/HAP transcription factor complex.
10 ^a^	28838402	6,10E-23	0,429	8,13	Intergenic, 2Kb upstream of blastx hit LOC_Os04g02960	NA	NA
9 ^a^	3379806	2,51E-10	0,479	5,94	Intergenic, 19Kb downstream of Phvul.009G018100 3351758.3360767	AT1G75900	GDSL-type esterase/lipase protein
1 ^a^	14629730	4,48E-17	0,059	4,10	Intergenic, 30Kb downstream of Phvul.001G089900 (14661480.14663300)	AT1G66350	DELLA protein, probable transcriptional regulator that acts as a repressor of the gibberellin (GA) signaling pathway.
10 ^a^	21557790	9,53E-15	0,091	4,04	Intergenic		
1 ^a^	20550800	5,94E-09	0,329	2,28	Intergenic Phvul.001G100800 (20556092.20556311) Phvul.001G100700 (20533891.20540720)	NA AT1G09230	NA RNA-binding (RRM/RBD/RNP motifs) family protein
1 ^a^	10461554	6,74E-08	0,093	1,28	Intergenic Phvul.001G075900 (10469774.10470233)	NA	NA
7 ^b^	24185078	8,70E-10	0,086	41,07	Phvul.007G146100 24184160.24187772	AT4G02425	LYR motif-containing protein 7
8 ^b^	13960516	8,32E-08	0,101	10,3	Phvul.008G116400 13963642.13964304	AT5G06060	Tropinone reductase homolog

a Sites associated to SG;

b sites associated to recovery.

NA, not available.

### Putative candidate genes

Most of the SNPs identified through GWAS were intergenic, except for one variant associated to recovery, and we therefore report the closest neighboring gene models on **Table 2**. We cannot discard however the possibility that these SNPs could be cis/trans-regulating other genes in the genome. The functional annotations of the closest gene models revealed interesting pathways that could be involved in the emergence of SG traits. For example, we identified elements from two important hormone signaling pathways, gibberellin and abscisic acid ABA, in chromosomes 1 and 8: PPR proteins have been associated to the response to drought, salt and cold stresses in *Arabidopsis* by negatively regulating ABA signaling pathways, while DELLA proteins are negative regulators of gibberellin signaling pathways. Furthermore, we identified a gene encoding an enzyme from the tocopherol (vitamin E) biosynthetic process, which is in turn an important antioxidant that protects thylakoid membrane lipids from photooxidation and helps plants cope with high light and heat stress (Niu et al., 2022). In chromosome 9 we identified a GDSL-type esterease/lipase protein, associated to drought tolerance in soybean (Su et al., 2020), and in chromosome 7 we found a gene model encoding a nuclear transcription factor Y subunit B-8. The two sites identified associated to recovery with MLMM encode a LYR motif-containing protein and a tropinone reductase homolog, which is involved in the synthesis of alkaloid compounds.

We then studied the gene models encoded within the chromosomic windows associated to each drought response strategy through F_ST_ outliers. We identified the gene model *Phvul.005G008300* (719,827-725,719bp) on chromosome 5, which is encoded in the most differentiated region between SG – E and between SG – S (F_ST_ >0.30). This gene model is annotated as a trehalose-phosphate-phosphatase A (TPPA), which removes the phosphate from trehalose 6-phosphate to produce free trehalose. When taken together, we did not find any significant GO enrichments, so we looked specifically for keywords associated to stay-green traits, such as photosynthesis, stress or senescence. With this search we found photosystem associated gene models on chromosomes 1, 5, 8, 9 and 10 (**Table 3**). In particular LHCA on chromosome 10 belongs to a family of proteins strongly associated to the stay-green phenotype in rice though the accumulation of chlorophyl (Yamatani et al., 2018). Within the one F_ST_ outlier of escaped accessions, we identified the upstream region of a riboflavin synthase, RISB, that catalyzes the formation of 6,7-dimethyl-8-ribityllumazine, which is the penultimate step in the biosynthesis of riboflavin. Another gene model associated to drought-escaping cultivars is Phvul.002G041400, encoding a PLATZ transcription factor.

**Table 3:** Photosynthesis related genes identified in Fst outlier windows in the SG subgroup.

Chr	Fst window start	Fst window end	Gene ID	Gene model start	Gene model end	overlap (bp)	Functional annotation
Chr01	50600001	50650000	Phvul.001G257100	50615288	50616052	764	AT1G67740.1 PSBY,YCF32 photosystem II BY
Chr05	1700001	1750000	Phvul.005G020200	1743797	1745863	2066	AT3G63540.1 Mog1/PsbP/DUF1795-like photosystem II reaction center PsbP family protein
Chr08	62950001	63000000	Phvul.008G292600	62969443	62970086	643	ATCG00680.1 photosystem II reaction center protein B
Chr09	32600001	32650000	Phvul.009G216400	32627625	32629139	1514	AT1G30380.1 PSAK photosystem I subunit K
Chr10	40250001	40300000	Phvul.010G121400	40260565	40262990	2425	AT3G61470.1 LHCA2 photosystem I light harvesting complex gene 2

## Discussion

One of the main constraints around the world for crop productivity is drought. To cope with this abiotic stress, it is necessary to understand the response mechanisms of plants that face scarce water conditions to improve yield (Huang et al., 2008). The common bean is highly sensitive to variations in temperature, humidity, and amount of nutrients (Schmit et al., 2019), hence unraveling the mechanisms behind drought tolerance is of utmost importance for its production.

Plants use various strategies to cope with drought, generally grouped into escape, avoidance (mostly in CAM plants), tolerance and recovery (Rosales-Serna et al., 2004). Drought tolerance involves a series of adaptations that allow a plant to withstand arid or drought conditions without affecting performance. These adaptations involve mechanisms to maintain turgor pressure through osmotic adjustment that includes an increase in the concentration of solutes, such as sugars, organic acids and ions. Increased cellular elasticity and decreased cell size due to protoplasmic resistance are also mechanisms contributing to drought tolerance (Bacelar et al., 2012; Azhar and Rehman, 2018). Drought escape relies on rapid reproduction before drought strikes. A successful reproduction involves a better partition of assimilates towards the seeds and fruits and the plant must therefore have the capacity to store reserves efficiently in organs, such as stems and roots, and be able to relocate them to produce fruits. This strategy has been widely seen in annuals and especially in ephemeral plants in desert environments (Bacelar et al., 2012). Drought recovery can be defined as the ability of the plant to recover after a period of drought. The mechanisms behind this strategy have not yet been elucidated, although studies in pea (*Pisum sativum*) suggest that the ability of nodulated plants to recover after drought could be explained by the re-launch of N acquisition and fine-tuning of nodule formation (Abid et al., 2018; Couchoud et al., 2020). Finally, although it has not been strictly considered among drought adaptation strategies, delayed senescence seems to play an important role in drought tolerance as well (Sekhon et al., 2019). In SG plants there is a delay in senescence caused by the impaired degradation of chlorophyll, contrary to what occurs in normal genotypes. This strategy maintains the leaves photosynthetically active and thus can positively influence the subsequent filling of the grain even under stress conditions. There are two types of SG genotypes, functional and cosmetic. In the cosmetic SG phenotype the plant retains chlorophyll but its photosynthetic capacity is lost (Thomas and Ougham, 2014). A functional SG occurs when photosynthesis proceeds normally for a prolonged period. Two variants of functional SG can be seen, type A, where the onset of senescence is delayed, while in type B, senescence begins normally but the process is slowed down (Kamal et al., 2019). Only a few common bean cultivars have been identified as SG, such as BRS Expedito, FT-Tarumã and BAF071 and these have been correlated with a lower incidence and severity of plant pathology, greater stem diameter, and higher grain yield (Schmit et al., 2019).

In general, dry beans are more sensitive to terminal drought, i.e. during the pre-flowering and flowering stages, causing an excessive abortion of flowers, young pods and seeds (Singh, 2007). At this point, reported genetic markers associated with drought tolerance in common beans are limited. Mukeshimana and collaborators (Mukeshimana et al., 2014) found QTLs for days to flowering and maturity located on chromosome 1 in plants subjected to drought stress. Recent work in a recombinant population of common beans found QTLs for pod harvest index, yield under drought stress conditions, highlighting its importance in the remobilization of photosynthates (Berny Mier et al., 2019). Asfaw et al. (Asfaw et al., 2012) found QTLs for traits related to drought tolerance, suggesting that the fraction of photosynthates remobilized from pods to seed is related to plant performance both under stress and non-stress conditions. Other works have used SNP-type molecular markers in recombinant inbred populations for the construction of linkage maps where several QTLs have been associated with yield traits in response to drought stress (Mukeshimana et al., 2014; Elias et al., 2021). Hoyos-Villegas et al. (Hoyos-Villegas et al., 2017) performed a GWAS analysis using ~6kSNPs on a panel of various bean genotypes native of Central America that were selected based on their previously described tolerance to drought. They found associations to a number of traits related to biomass, seed weight, and wilting that may be involved in drought resistance. At the transcriptional level, Pereira et al. (Pereira et al., 2020) analyzed the response to drought in common bean roots and leaves, contrasting the genotypes BAT477 and Pérola which are resistant and susceptible to drought, respectively.

In this report we decided to evaluate the response to terminal drought at the flowering stage under greenhouse conditions on a collection of photoperiod insensitive *P. vulgaris* cultivars aimed at covering the natural variation of the species. As expected, several accessions could not survive the lack of water or had severe yield loss of >75%; these were considered susceptible cultivars to drought (22 in total). Although photoperiod sensitivity did not allow us to evaluate wild accessions from the Americas during the spring-summer seasons at Scandinavian latitudes, we could identify three drought tolerant accessions, G3296 (MA), G12875 (MW) and G23458 (AW), in climatic chambers (at neutral day-length, data not shown) which gave us an indication that resistance is not associated with a particular gene pool. Based on the leaf and shoot greenness during the experiment, which evidently encompasses many physiological processes beyond late senescence (Pinto et al., 2016), a total of six accessions were classified as SG, despite the differences in yield gain or loss. We also identified 16 drought escaping cultivars and 17 recovered.

The onset of foliar senescence depends mainly on the ontogeny of the plant. However, this process can be induced prematurely to accelerate the remobilization of nutrients in response to environmental changes, such as biotic or abiotic stress conditions. This process provides enough energy to start the reproductive stage, especially important in annual species, to complete their life cycle and generate offspring (Bengoa Luoni et al., 2019). This was observed in most of the screened accessions (**Figures 1A, B**) that tried to accelerate their reproductive process by increasing pod production, especially during the first week of treatment, although in many cases the pods were aborted or not filled with seeds. The opposite was observed in the SG genotypes, in which development was not interrupted, just slowed down while water was scarce. The fact that these plants could be harvested, even with differences in yield loss, suggests a functional SG phenotype, probably type A.

### Genetic basis of stay-green phenotype and other drought responses

SG traits have been identified in various crops as key components in breeding to increase yield and stress tolerance to drought and salinity. The advantages provided by delayed senescence have been previously reported in model species such as *Arabidopsis thaliana* (Wingler et al., 2012) and in some cereals (Fahad et al., 2017), where a greater capacity to tolerate abiotic stress as high temperatures and drought in green genotypes was identified. Furthermore, transgenic tobacco plants where drought-induced leaf senescence was suppressed, display outstanding drought tolerance and minimal yield loss (Rivero et al., 2007). This increased tolerance results from the protection of photosynthetic structures against reactive oxygen species, such as superoxide and peroxide (Rivero et al., 2007; Thomas and Ougham, 2014). Also, the relationship between senescence and stress caused by drought in plants became evident when studies on multi-parent advanced generation inter-cross (MAGIC) wheat lines indicated that, in general, in all lines the onset of senescence can be predicted from the plant water consumption (Camargo et al., 2018).

The most visible change during leaf senescence is associated to chlorophyll degradation and the decay of photosynthetic capacities. However, in SG plants the greenness of the leaves remains longer and when functional, SG traits allow the plant to photosynthesize for longer and have higher yields. Different proteins involved in chlorophyll degradation have been studied in rice and wheat, showing differential accumulation in SG cultivars. For example, a rice mutant *delayed yellowing 1* (*dye1*) accumulates higher amounts of chloropyll l than the wild-type in pre-senescent leaves. Positional cloning revealed that the *DYE1* gene encodes *Lhca4*, a subunit of the light-harvesting complex I (LHCI) (Yamatani et al., 2018). Furthermore, a wheat SG mutant, *tasg1*, exhibits a delayed senescence and slow degradation of chlorophyll. In a study of the stability of proteins in thylakoid membranes under drought stress, the authors observed that, compared to the wild type, in *tasg1* plants the expression levels of *Lhcb4* and *6* were higher; the abundance of some polypeptides in thylakoid membranes was greater and the accumulation of superoxide radicals and hydrogen peroxide was lower. These results suggested greater functional stability of the thylakoid membrane proteins, and higher antioxidant competence of *tasg1* to respond to drought stress (Tian et al., 2013). In the case of common bean, a comparative proteomic study on isolated chloroplasts from leaves of two cultivars under drought stress revealed that 44 proteins changed abundance between control and stressed plants. The majority of them were involved in photosynthetic processes (Zadraznik et al., 2019). In this study we identified several gene models with functional annotations related to photosynthetic activity and thylakoid stability (**Table 3**) encoded within genomic windows that differentiate SG cultivars.

On chromosome 7 we identified the gene model *Phvul.007G134000*, located 10.3Kb away from a strong GWAS signal. This model is annotated as a *nuclear transcription factor Y subunit B-8* (*NFYB8*). Several studies have associated the NF-Y complex to stress response in plants, and NF-YB has been studied as a regulator of drought stress in soybean (Sun et al., 2022), where its overexpression enhanced drought resistance, yield accumulation, less leaf damage and high superoxide dismutase concentration compared with control plants, to help scavenge the oxygen free radicals. In maize, transgenic plants under water limitation with increased *ZmNF-YB2* expression show tolerance to drought based on the responses of a number of stress-related parameters (like chlorophyll content, stomatal conductance, leaf temperature, reduced wilting, and maintenance of photosynthesis) that contribute to a grain yield advantage (Nelson et al., 2007).

Another relevant SG candidate gene is located on Chr05, *Phvul.005G008300*, and although no significant GWAS signal was found within the gene, it is encoded in the most differentiated region according to the F_ST_ pairwise comparisons. The orthologue of this gene in *A. thaliana, AT5G51460*, is annotated as a *trehalose-phosphate phosphatase A* (*AtTPPA*) that removes the phosphate from trehalose 6-phosphate to produce free trehalose (Ponnu et al., 2011). The accumulation of this non-reducing disaccharide improves abiotic stress tolerance, as it has been reported to have a function in stabilizing proteins against denaturation and acts as an osmoprotectant in the maintenance of cellular osmotic balance (Singh et al., 2015). Under dehydration conditions, trehalose plays a role in stabilizing dehydration-related enzymes and proteins, as well as lipid membranes, and it can scavenge ROS to protect biological structures from damage. Given that the concentration of trehalose in the cell is very low (approximately three orders of magnitude lower than sucrose), small changes in its concentration can lead to large changes in stress tolerance compared to other sugars (Lin et al., 2019). *Trehalose-6-phosphate synthase* (*TPS*) and *trehalose-6-phosphate phosphatase* (*TPP*) are the catalysts in the synthesis of trehalose. As reviewed by Oladosu et al. (Oladosu et al., 2019), the expression of a fusion *TPP*/*TPS* gene from *E. coli* in rice, resulted in a higher concentration of trehalose and better resistance to drought and less photooxidation to salt stress. Similarly, in *A. thaliana*, the loss-of-function mutation of a *trehalose-6-phosphate phosphatase* (*TPP*), resulted in a drought- sensitive phenotype, while overexpression of the gene triggered a significantly increased drought tolerance and trehalose accumulation (Lin et al., 2019).

The strongest gene candidates behind the drought escape strategy were identified on chromosome 2. Phvul.002G041400, a model encoding a Plant AT-rich sequence and zinc binding (PLATZ) transcription factor, belongs to a class of plant specific zinc-dependent DNA-binding proteins that function in abiotic stress response and development. In soybean, *GmPLATZ17* has been reported as a suppressor of drought tolerance by interacting with *GmDREB5*, interfering with its ability to bind its target genes and thereby, regulating drought stress response (Zhao et al., 2022). Furthermore, in a different region of chromosome 2 we identified RISB that participates in the biosynthesis of riboflavin (Uniprot). RISB interacts with *SINA6* (IntAct, 3 detection methods, score 5.6), a probable inactive E3 ubiquitin-protein ligase that plays a role in regulation of autophagy, and acts as positive regulator of drought stress response by positively regulating abscisic acid-mediated stomatal closure.

## Conclusion

Drought response is a multi-genic, intricate phenotype. More analyses will be needed to measure the cellular responses behind delayed senescence in the SG common bean cultivars, however we have produced a good overview of the genomic regions that could be playing major roles in the emergence of SG traits in response to drought stress. As the SG accessions were only identified in the European gene pool, we need to extend our screening among American accessions to assess if these traits emerged recently and thus, would be good candidates for introgression into other domesticated cultivars.

## Data availability statement

The dataset presented in this study can be found in online repositories, through NCBI/SRA bioproject PRJNA1004188.

## Author contributions

MR-A and PI planned and designed the research. DL and MR-A performed experiments and analyzed data. MR-A wrote the manuscript with input from DL and PI. All authors read and approved of the final version of the manuscript.
