# Supplementary material for: Dissecting the genetic basis of drought responses in common bean using natural variation
Source: Front Plant Sci. 2023 Sep 14;14:1143873. doi: 10.3389/fpls.2023.1143873 (PMC10538545; doi:10.3389/fpls.2023.1143873)
Supplement: Supplementary file 1 [file DataSheet_1.docx]

**Supplementary figures**


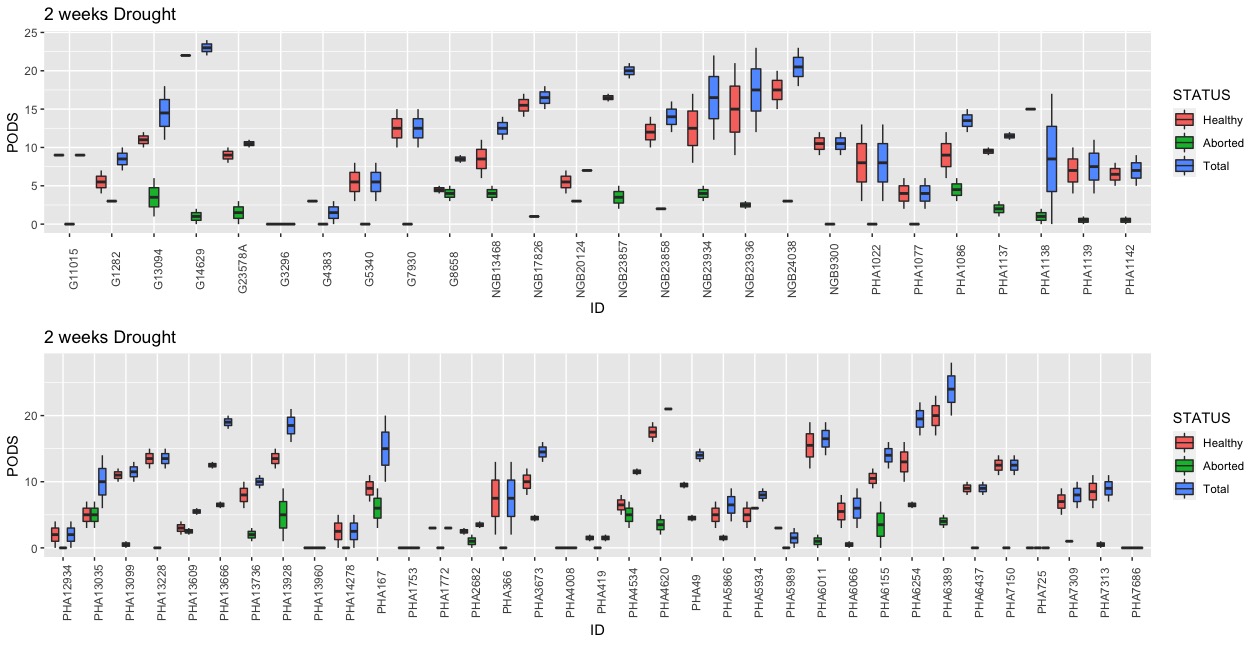


**Supplementary figure 1.** Number of pods, filled and aborted, per accession after two weeks of drought stress.

**
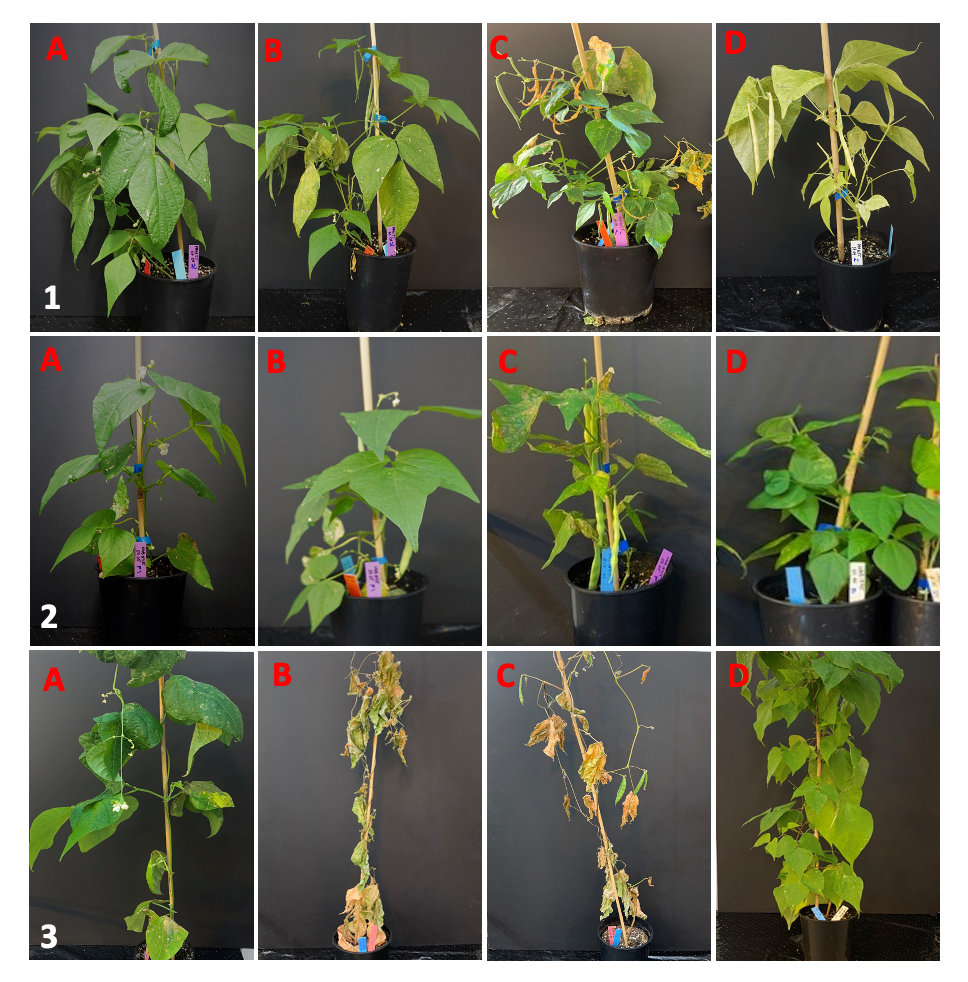
**

**Supplementary figure 2.** Phenotyping summary. Accessions PHA6155 (1, SG), NGB18415 (2, SG) and PHA12934 (3, susceptible) during the treatment under green-house conditions; the panels are labeled as follows: A, 1 WOT, B, 2 WOT, C, 1 WAR. D: Control [WOT=weeks of treatment, WAR=Weeks after re-watering].

**Supplementary figure 3**. Population structure. SNP-based PCA of the phenotyped cultivars (126,111 pruned sites across the 11 chromosomes). Left side colored by gene pool and right side colored by drought response strategy. Genepool: A=Andean, AW=Andean wild, EU= European, MA=Mesoamerican, MW=Mesoamerican wild. Strategy: E=escape, SG=stay-green, R=recovery and S= susceptible**.**
